# Fevers

**Published:** 1905-07-29

**Authors:** 


### FEVERS.

**Plague.**—The prevalence of plague in India[1] not only continues, but increases. In 1903 the recorded deaths from this cause numbered 853,000; in 1904 the figures rose to 1,040,000. Of these million odd deaths 350,000 occurred in one province, the Punjab, which furnishes out of its population of about 20,000,000 some of the best native Indian soldiers. Of this number 250,000 were swept off in the space of twelve weeks only. Taking the returns for the whole of India, the Secretacy of State computes that over 200,000 deaths from plague occurred in January and February of the present year, while the deaths since the outbreak in 1896 total three and a half millions. Two bacteriologists have been officially despatched to study the disease in view of this great mortality. The whole subject of plague, especially in its relation to India, is dealt with in a treatise recently published by Professor Simpson.[2] A review of the history of the disease shows how difficult its eradication has been in years gone by. Even in ancient times disease and death in rats and mice have always been associated with plague in man, and it has been suggested by Gotschlich and others that the apparently seasonal falls and rises in virulence of the disease may be due to latent or chronic forms of plague in the older rats, alternating with more virulent outbursts in the younger animals. Many pressing ætiological problems await solution regarding the mode of transmission from rats to man, and the part played by fleas and other vermin. Meanwhile the machinery for checking the disease in India is wholly inadequate, and has been abandoned as hopeless, and plague stalks the land practically unhindered and unlimited. A careful study of the outbreak in Natal,[3] 1902–3, has been made by the health officer, Dr. Hill, who concludes that there is no reason to question the agency of the rat. An experiment was made to determine how long plague germs would remain virulent in infected grain. Healthy rats were introduced at varying times after the death of plague-stricken rats in the same granary. Those introduced four weeks after the last plague-stricken rat had died sickened, while those introduced after two months remained healthy. Hence it is concluded that the plague organisms rapidly lose virulence after four to eight weeks' separation from a living host. The part played by insects in carrying plague germs is not yet determined. Herzog[4] in an article dealing with the subject, presents the views of a considerable number of observers in different parts of the world. Most of these are disposed not to believe in the intermediation of suctorial insects, and especially of fleas, and many instances are adduced where those in attendance on plague patients or making post-mortems on the bodies of such, although bitten by fleas which had infested these, have escaped. Even if fleas do take the bacilli into their bodies it does not follow they can transmit them to their next host. Nor is it proved that rat-fleas are identical with those infesting human-kind. Herzog suggests that pediculi capitis may perhaps convey the disease and cites at length one case in support of this idea, that of a native child in Manila in whom pediculi capitis were found and in whom the primary bubo occurred in the cervical glands.

**Malta Fever.**—The first report of the Commission appointed, at the request of the Colonial Secretary, by the Royal Society for the investigation of this scourge of our naval and military forces in the Mediterranean has been recently published. The researches of Major Horrocks[5] and his co-workers were chiefly directed to determining the vitality of the micrococcus melitensis outside the body in soil, fabrics, etc., under varying conditions. The organism was found to be highly resistant, a fact which would account for its ready dissemination by means of dust and the consequent probability of infection through the buccal-nasal passages. Hitherto experiments have failed to show that the mosquito acts as an intermediate host.

[1] Lancet, May 6, p. 1211. [2] Ibid., p. 1205. [3] Brit. Med. Jour., March 18, p. 635. [4] Amer. Jour. Med. Sc., March. [5] Brit. Med. Jour., April 15, p. 839.

